# Prognosis and risk stratification in first-presentation myocardial infarction with nonobstructive coronary arteries using stress cardiac MRI

**DOI:** 10.1186/s13244-026-02316-2

**Published:** 2026-05-29

**Authors:** Leting Tang, Qian Long, Wenjin Zhao, Kang Li, Lin Tian, Hu Guo, Haiyang Li, Mu Zeng

**Affiliations:** 1 Department of Radiology, The Second Xiangya Hospital, Central South University, Changsha, China; 2 Circle Cardiovascular Imaging Inc., Changsha, China; 3 MR Application, Siemens Healthineers Ltd., Changsha, China; 4 Clinical Research Center for Medical Imaging in Hunan Province, Changsha, China

**Keywords:** MINOCA, Cardiac magnetic resonance, Stress perfusion, Late gadolinium enhancement, Prognosis

## Abstract

**Objectives:**

To evaluate the prognostic value of stress cardiac MRI in myocardial infarction with nonobstructive coronary arteries (MINOCA), and to investigate its efficacy in risk stratification.

**Materials and methods:**

A total of 279 MINOCA patients undergoing stress cardiac MRI were retrospectively enrolled. Patients were classified into 4 subgroups according to the cardiac MRI phenotype: (1) presence of late gadolinium enhancement (LGE) and inducible ischemia+ (*n* = 95); (2) LGE+/inducible ischemia− (*n* = 72); (3) LGE–/inducible ischemia+ (*n* = 69); and (4) LGE–/inducible ischemia− (*n* = 43). The primary outcome was major adverse cardiovascular events (MACE).

**Results:**

Over a median follow-up of 41 months, MACE occurred in 11.5% of patients. Those who developed MACE showed significantly reduced cardiac function (left ventricular ejection fraction (LVEF): 38.6 [35.2; 51.6] vs 54.5 [48.7; 61.6], *p* < 0.001), severe microvascular dysfunction (myocardial perfusion reserve (MPR): 1.5 [1.4; 1.7] vs 1.9 [1.8; 2.6], *p* < 0.001), and extensive myocardial damage (LGE: 9.6 [7.7; 25.6] vs 2.4 [0.0; 6.2], *p* < 0.001). Multivariate Cox regression showed that LGE (HR: 1.108, 95% CI: 1.047–1.172, *p* < 0.001) and MPR (HR: 0.062, 95% CI: 0.007–0.524, *p* = 0.011) were independently associated with MACE. Kaplan–Meier analysis indicated that patients with LGE ≥ 8.39 and MPR < 1.75 had a significantly higher MACE risk (*p* < 0.001).

**Conclusion:**

In MINOCA patients, LGE and MPR independently predict MACE risk. Particularly, MPR offers incremental prognostic value by further stratifying patients with limited LGE.

**Critical relevance statement:**

MINOCA is a clinically important and heterogeneous entity, often associated with adverse long-term outcomes. Stress cardiac MRI parameters, LGE and MPR, can predict the prognosis of patients with MINOCA, facilitating risk stratification and guiding subsequent management decisions.

**Key Points:**

- Stratifying MINOCA patients into subgroups based on cardiac MRI tissue and functional characteristics enables refined risk stratification.
- In MINOCA patients with limited LGE, impaired MPR is associated with a higher incidence of MACE.
- Microvascular dysfunction may be recognized as one of the potential mechanisms contributing to MINOCA and is associated with adverse cardiovascular outcomes.

**Graphical Abstract:**

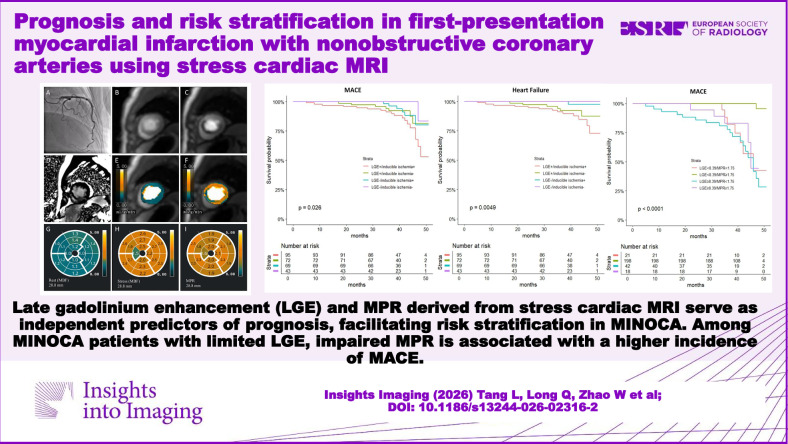

## Introduction

Myocardial infarction with nonobstructive coronary artery (MINOCA) is characterized by acute myocardial infarction (MI) where angiography reveals no obstructive coronary artery disease (stenosis severity < 50%), in the absence of a clinically overt alternative cause (e.g., systemic causes of type 2 MI like sepsis, pulmonary embolism, hypertension, myocarditis and arrhythmias) [1]. The estimated prevalence of MINOCA ranges from 5% to 15% among all patients with acute MI [2–6]. According to the American Heart Association scientific statement in 2019, MINOCA is caused by a large and heterogeneous group of etiologies, including coronary artery plaque disruption, coronary vasospasm, spontaneous coronary artery dissection, coronary embolism/thrombosis, and coronary microvascular dysfunction [7].

Cardiac MRI is recommended in European Society of Cardiology (ESC) guidelines when the final diagnosis of MINOCA remains uncertain [8]. Cardiac MRI can accurately identify the presence, extent, and pattern of myocardial edema, inflammation, scar, and fibrosis, and simultaneously analyze the cardiac structure and function. In addition, quantitative stress cardiac MRI can detect microvascular dysfunction as reduced stress myocardial perfusion reserve [9, 10]. An observational study demonstrated that cardiac MRI could identify a final diagnosis in 74% of patients presenting with MINOCA [11].

Clinical outcomes in MINOCA vary depending on the underlying cause [12–14]. The prognosis of MINOCA is not as benign as previously thought [15]. A recent meta-analysis revealed a 1-year mortality rate of 4.7% (95% confidence interval (CI): 2.6–6.9%) among these patients [3]. Approximately 25% of MINOCA patients experienced major adverse cardiovascular events (MACE) within 4 years [5, 16].

Despite the recognized diagnostic utility of cardiac MRI in MINOCA, its utility for subsequent risk stratification based on stress findings remains inadequately explored. This study, therefore, aimed to investigate the prognostic value of stress cardiac MRI parameters in MINOCA, with the goal of identifying quantifiable markers that could refine risk stratification and guide management of this clinically heterogeneous condition.

## Materials and methods

### Study design and population

This study was designed as a retrospective, observational cohort study to evaluate prognostic information in patients admitted to the Second Xiangya Hospital between January 2021 and December 2023 with a clinical diagnosis of MINOCA who were referred for cardiac MRI assessment. Inclusion criteria followed current American Heart Association guidelines [7], which encompassed the following: (1) the presence of acute MI, defined as a rise or fall in cardiac troponin level above the 99th percentile upper reference limit, along with supportive clinical evidence of infarction according to the 4th universal definition of MI [17], and (2) the absence of notable coronary artery obstruction on coronary angiography (stenosis severity < 50%). This study was approved by the ethics committee of the Second Xiangya Hospital of Central South University (LYF2023027), and informed consent was provided by all participants.

In accordance with guideline recommendations, all patients with suspected MINOCA were evaluated using a diagnostic algorithm designed to distinguish ischemic MINOCA from non-ischemic mechanisms [7, 8, 18]. Coronary angiography was performed during the acute phase in all patients, who also underwent comprehensive electrocardiography and transthoracic echocardiography. The exclusion criteria were as follows: (1) troponin elevation due to pulmonary embolism, septicemia, myocardial contusion, and non-ischemic myocardial injury (i.e., myocarditis and Takotsubo cardiomyopathy); (2) patients with a prior history of acute myocardial infarction; (3) previous coronary stenting or revascularization, pregnancy; (4) patients with MINOCA having contraindications to cardiac MRI and coronary angiography; (5) poor MR image quality. The diagnosis of myocarditis was considered based on: (1) a history of recent prodromal infection or autoimmune disease, along with clinical symptoms and signs consistent with myocarditis; (2) laboratory evidence of viral nucleic acids (e.g., coxsackievirus, adenovirus) or significantly elevated viral antibody titers; and (3) cardiac MRI findings consistent with the Lake Louise Criteria, most commonly showing subepicardial or mid-myocardial late gadolinium enhancement. The diagnosis of Takotsubo syndrome was considered based on the following: (1) a preceding emotional, physical, or combined trigger; (2) echocardiographic evidence of transient left ventricular dysfunction (hypokinesia, akinesia, or dyskinesia) presenting as apical ballooning or midventricular, basal, or focal wall motion abnormalities, with the regional wall motion abnormality usually extending beyond a single epicardial vascular distribution; and (3) the absence of significant late gadolinium enhancement in cardiac MRI. In addition, all patients’ ECG monitoring records were reviewed to exclude arrhythmia as a potential trigger of myocardial injury. All patients were hospitalized and received guideline-directed medical therapy for MINOCA. Clinical data were collected from hospital records.

The institutional ethics committee of Central South University’s Second Xiangya Hospital approved the study protocol, and all participants provided written informed consent.

### Cardiac MRI and imaging acquisition

Cardiac MRI examinations in this study were performed during the non-acute phase of MINOCA. The interval from acute presentation to cardiac MRI ranged from approximately 1 to 3 months. A subset of patients experienced transient, mild side effects, such as palpitations. No serious adverse events occurred during or immediately following any stress test in our cohort.

**Fig. 1:**
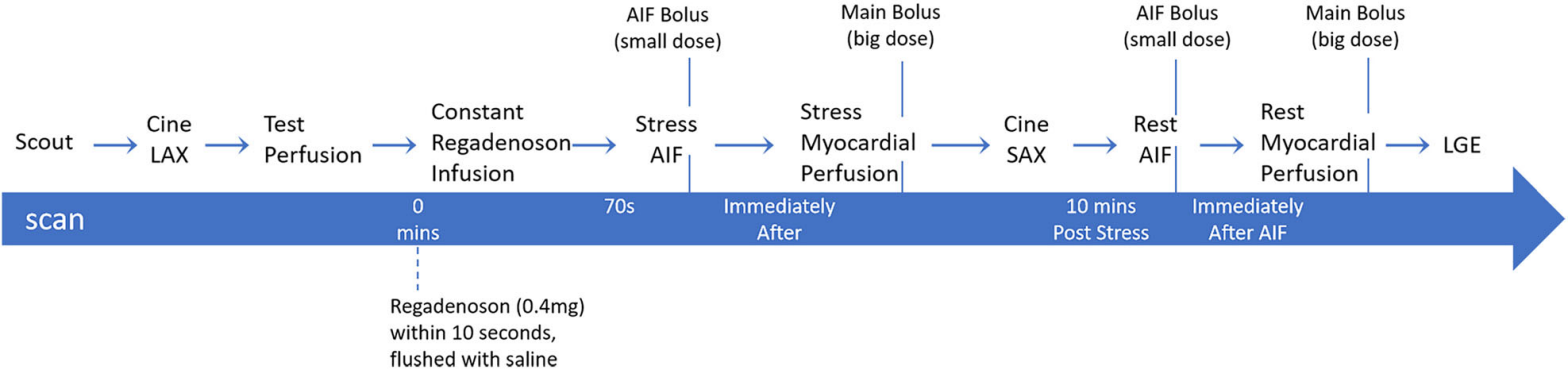
The stress cardiac magnetic resonance scanning protocol. AIF, arterial input function; LAX, long-axis; LGE, late gadolinium enhancement; SAX, short-axis

All participants underwent cardiac MRI on a 3.0-T scanner (MAGNETOM Skyra; Siemens Healthineers) using an 18-channel body coil. An electrocardiogram was used to calibrate myocardial blood flow (MBF) measurements at rest and stress based on heart rate. Myocardial perfusion imaging was performed utilizing a steady-state free precession pre-bolus technique for estimating absolute MBF [19]. Regadenoson (0.4 mg) was administered as an intravenous injection within 10 s and flushed with saline. After a 70-s waiting stress arterial input function (AIF) imaging was initiated, followed by a small-dose AIF bolus (0.0075 mmol/kg, calculated from body weight) with a subsequent saline flush of at least 20 mL, continuing for a total of 60 frames. Immediately after, stress myocardial perfusion acquisition was commenced under free-breathing. After about five baseline frames, the main bolus was injected at a big dose (0.075 mmol/kg, calculated from body weight; please see the website for further calculation information: https://www.circlecvi.com/qp-protocol/index.html), with a subsequent at least 20 mL saline injection. Continuous stress myocardial perfusion acquisition was obtained for a total of 60 frames. During the subsequent 10-min waiting period, short-axis (SAX) cine imaging is performed. After 10 min, rest imaging is conducted during free breathing, similar to the stress protocol. After completing rest myocardial perfusion imaging, late gadolinium enhancement (LGE) images were acquired. The scanning protocol is shown in Fig. 1.

Myocardial perfusion imaging was performed using a saturation recovery sequence based on a fast low-angle shot imaging sequence, along with 3 myocardial short-axis slices of the left ventricle (basal, mid, and apical). The scan parameters were: repetition time = 2.0 ms; echo time = 1.0 ms; flip angle = 10°; field of view = 320 × 400 mm; acquisition matrix = 125 × 256; slice thickness = 8 mm and slice gap = 12 mm; temporal resolution = 110 ms.

Breath-hold cine imaging was performed using a segmented balanced steady-state free precession (bSSFP) sequence. The scanning parameters were as follows: repetition time = 3.2 ms; echo time = 1.43 ms; flip angle = 44°; temporal resolution = 40 ms; field of view = 320 × 400 mm; acquisition matrix = 126 × 224; slice thickness = 8 mm with 2 mm gap.

The standard technique for two-dimensional (2D) LGE acquisition is a segmented phase-sensitive inversion recovery (PSIR) and bSSFP sequence with breath-holding. Scan parameters were as follows: repetition time = 2.8 ms; echo time = 1.3 ms; flip angle = 40°; field of view = 320 × 400 mm; acquisition matrix = 125 × 256.

### Cardiac MR imaging analysis

All cardiac MR images were analyzed using the commercial post-processing Circle software (Circle Cardiovascular Imaging Inc.). The left ventricular ejection fraction (LVEF), end-systolic volume index (ESVi) and end-diastolic volume index (EDVi) were calculated from the cine images by two experienced radiologists.

LGE was identified as a myocardial gray threshold higher than the average signal intensity of the normal myocardium by five standard deviations. A normal myocardial region of interest was delineated far from the LGE region. The software automatically calculated the total enhanced volume as a percentage of the left ventricular myocardium volume. The presence and patterns of LGE were collected and considered as transmural if involved > 50% of the wall thickness. Inducible ischemia was defined as the presence of a perfusion defect under stress in a region without LGE. This assessment was qualitatively assessed by radiologists.

According to the American Heart Association, left ventricular segmental analysis, the myocardium was divided into 16 segments [20]. The software provided a fully automated framework that includes a model-constrained deconvolution technique to estimate pixel-wise MBF values in mL/min/g by processing time-signal intensity curves [19]. The software automatically delineated the endocardial and epicardial boundaries, with manual corrections applied as necessary. Myocardial perfusion reserve (MPR) was calculated as stress MBF divided by rest MBF [9].

Based on cardiac MRI findings, MINOCA were classified into 4 phenotypes: (1) LGE+/inducible ischemia+: an ischemic subendocardial or transmural LGE pattern with concomitant stress-induced perfusion defects; (2) LGE+/inducible ischemia−: presence of LGE without evidence of stress-induced perfusion defects; (3) LGE−/inducible ischemia+: cases without LGE but exhibiting regional myocardial ischemia; (4) LGE−/inducible ischemia− (normal cardiac MRI): MINOCA cases with stress cardiac MRI showing no LGE or inducible myocardial ischemia. To ensure diagnostic consistency, all cardiac MR images were given to two qualified radiologists for blinded review. In cases of disagreement, a third senior radiologist was adjudicated to make the final diagnosis.

### Study endpoint

The primary clinical endpoint was major adverse cardiovascular events (MACE), including all-cause mortality, reinfarction, stroke, and heart failure. For the MACE calculation, the first event was taken into account. Secondary outcomes included the individual components of MACE. All-cause mortality referred to death from any cause that occurred during follow-up. Stroke was characterized as an ischemic cerebral infarction resulting from embolic or thrombotic occlusion of a major intracranial artery. Reinfarction and heart failure were diagnosed in accordance with the recent ESC guidelines [17, 21]. Patients were followed up until June 30, 2025, by reviewing digital medical records, with supplementary telephone calls made to ensure completeness.

### Statistical analysis

Normal distribution was assessed by the Shapiro–Wilk test. Continuous variables with normal distribution were described as mean ± standard deviation, whereas those with non-normal distribution were described as medians and interquartile ranges. Student’s *t*-test, Mann–Whitney U test, ANOVA or Kruskal–Wallis tests were used to compare continuous variables between groups, as appropriate. Categorical data were displayed as an absolute value (percentage), and the chi-square test or Fisher’s exact test was used for comparison, as appropriate. Clinical outcomes were estimated using the Kaplan–Meier method and compared with the log-rank test. Data from patients lost to follow-up were censored at the time of their last contact. Cox proportional hazards methods were applied to identify predictors of MACE. Prognostic variables were first selected using the least absolute shrinkage and selection operator (LASSO) algorithm, and those identified were subsequently incorporated into a multivariable model via forward stepwise selection, with *p*-values set at 0.05 for entry and 0.10 for removal. Based on the median risk score derived from the predictive model, patients were stratified into high- and low-risk groups. We evaluated proportional hazard assumptions using the Schoenfeld residuals. Patients were stratified into four groups using cutoff values derived from the receiver operating characteristic (ROC) analysis, and their survival was compared using Kaplan–Meier curves. The reproducibility of cardiac MRI parameter measurements was analyzed using the intraclass correlation coefficient (ICC). Post hoc power analysis was conducted using PASS software (v21.0.3). All statistical analyses were performed using SPSS 26.0 (IBM Corp.) and R (version 4.5.1, The R Project for Statistical Computing).

## Results

### Patients’ characteristics

**Fig. 2:**
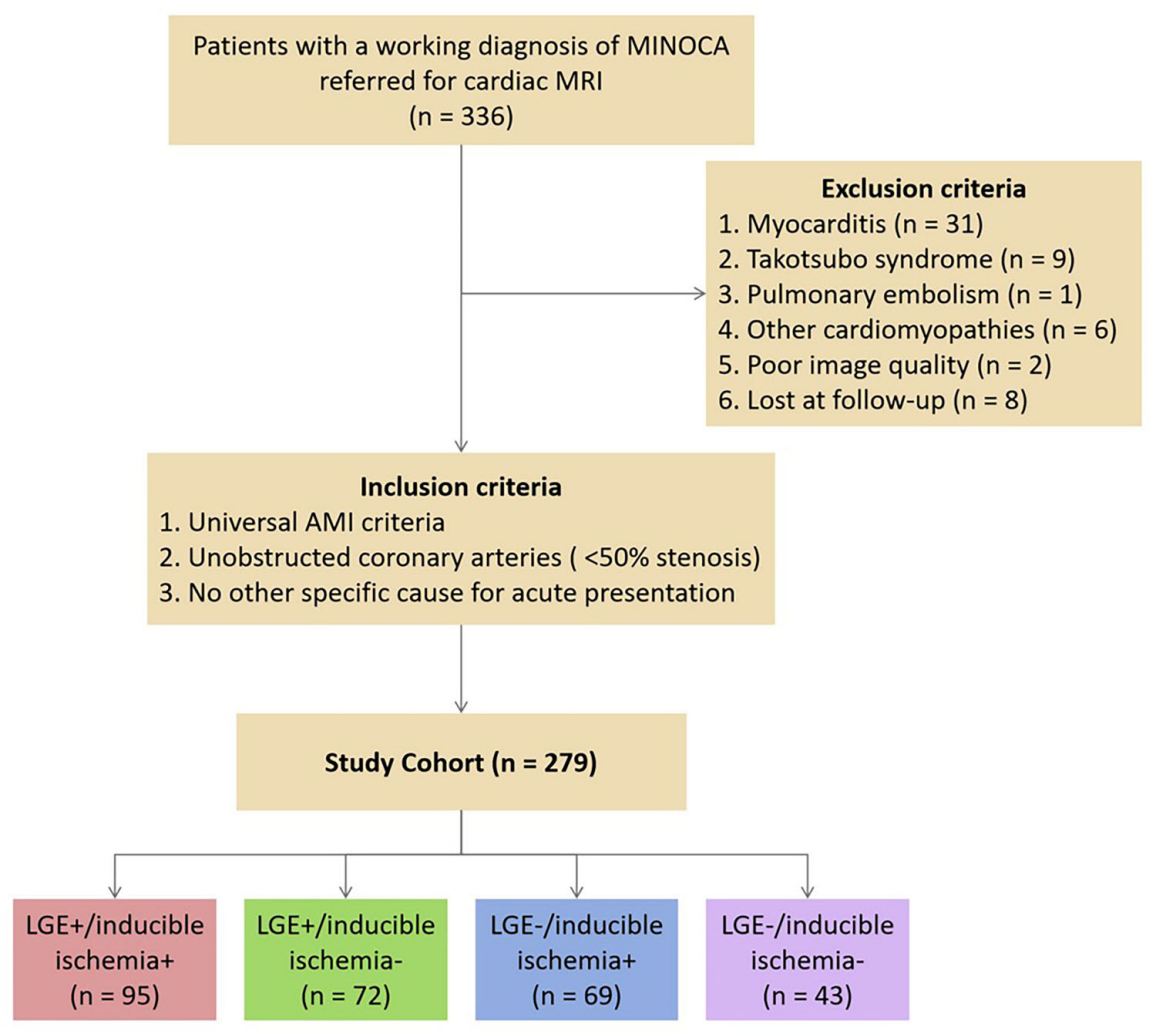
Study flowchart. LGE, late gadolinium enhancement; MINOCA, myocardial infarction with nonobstructive coronary arteries; MRI, magnetic resonance imaging

**Fig. 3:**
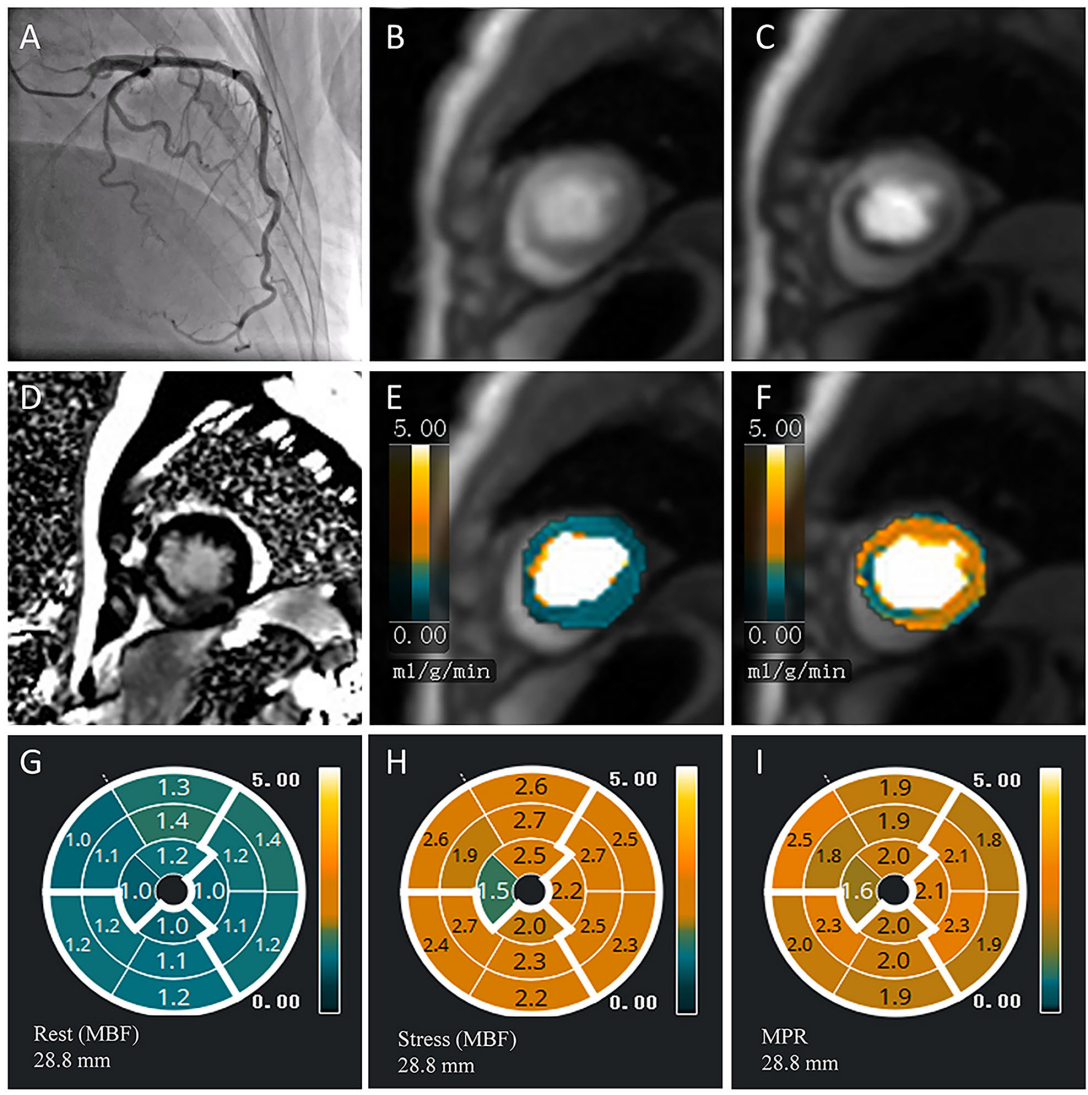
Representative case of a patient with stress perfusion defect. **A** Coronary angiography revealed no obvious stenosis in the left anterior descending artery. **B** Perfusion defect was not present in the rest perfusion image. **C** Inducible perfusion defect was observed in the stress perfusion image. **D** LGE was present in all left-ventricular walls on short-axis views. **E**, **F** Rest and stress MBF pixel maps. Stress perfusion defects are observed and are larger in extent than the corresponding subendocardial infarct within the same vascular territory. **G**–**I** The bull’s eye map of quantitative myocardial perfusion showing the rest MBF, stress MBF and MPR. LGE, late gadolinium enhancement; MBF, myocardial blood flow; MPR, myocardial perfusion reserve

**Fig. 4:**
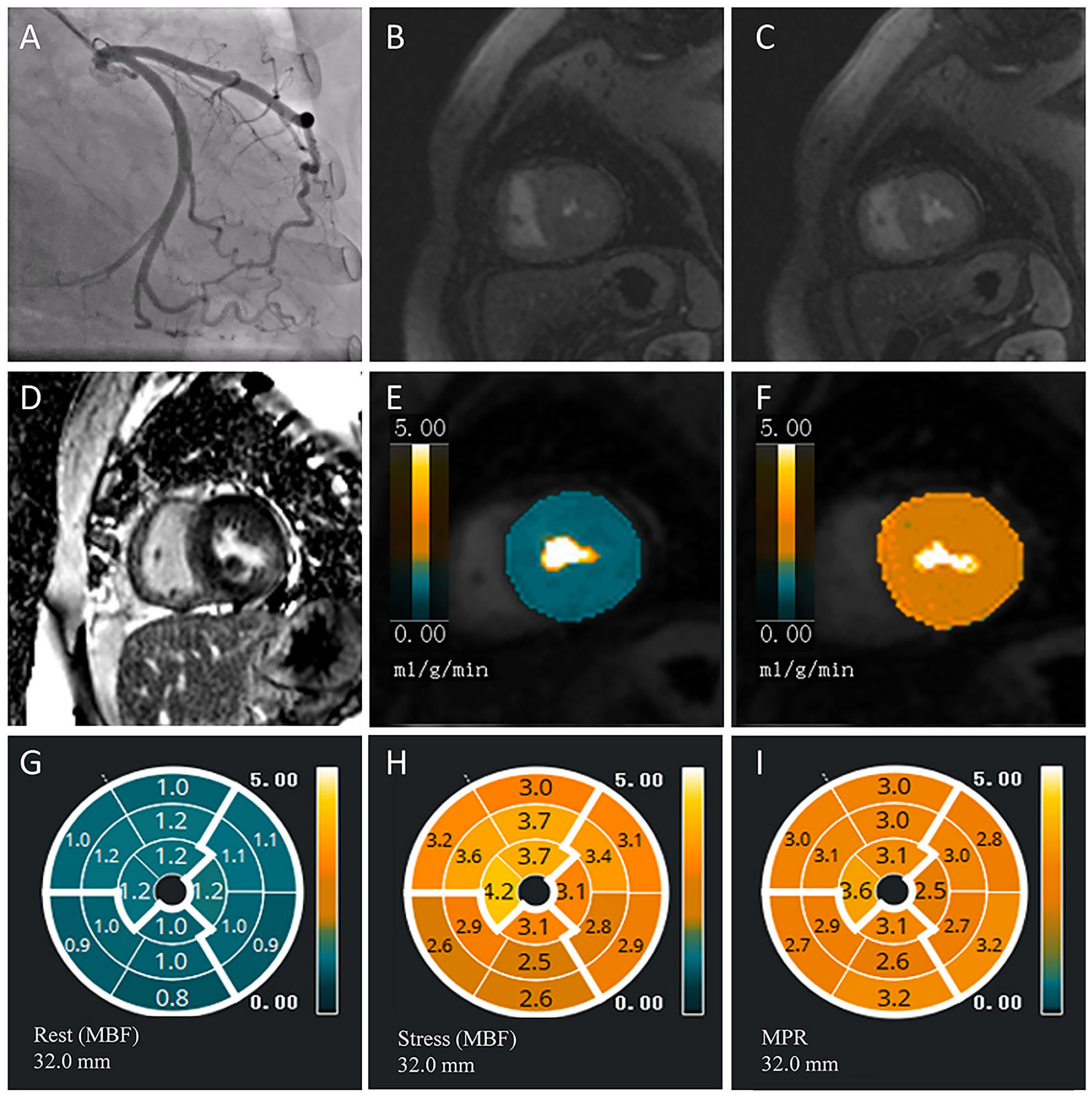
Representative case of a patient without stress perfusion defect. **A** No obvious stenosis in the left circumflex artery. **B**, **C** No visible perfusion defects in rest and stress perfusion images. **D** LGE was present in the lateral wall of the left ventricle. **E**, **F** Rest and stress MBF pixel maps. **G**–**I** The bull’s eye map of quantitative myocardial perfusion showing the rest MBF, stress MBF and MPR. LGE, late gadolinium enhancement; MBF, myocardial blood flow; MPR, myocardial perfusion reserve

During the inclusion period, among 336 individuals with a working diagnosis of MINOCA referred for stress cardiac MRI (Fig. 2). Of these, 57 were excluded and 279 were included in the final study population. Patients were divided into 4 subgroups according to cardiac MRI findings: (1) a total of 95 (34.1%) patients with LGE+/inducible ischemia+; (2) a total of 72 (25.8%) patients with LGE+/inducible ischemia−; (3) a total of 69 (24.7%) patients with LGE−/inducible ischemia+; and (4) a total of 43 (15.4%) patients with LGE−/inducible ischemia−. Baseline clinical characteristics and cardiac MRI data for the overall cohort and each subgroup are presented in Table 1. Representative cases of patients with and without stress perfusion defects are shown in Figs. 3 and 4.

**Table 1:** Baseline clinical characteristics and MRI parameters of patients with MINOCA according to cardiac MRI phenotypes

	Total (*N* = 279)	LGE+/inducible ischemia+ (*n* = 95)	LGE+/inducible ischemia– (*n* = 72)	LGE−/inducible ischemia+ (*n* = 69)	LGE−/inducible ischemia− (*n* = 43)	*p*-value
Age, years	56.35 ± 10.86	57.36 ± 9.64	55.72 ± 12.18	55.61 ± 11.23	56.37 ± 10.65	0.712
Female, %	154 (55.2)	51 (53.7)	41 (56.9)	39 (56.5)	23 (53.5)	0.964
BMI, kg/m^2^	26.03 ± 4.32	25.71 ± 4.40	26.22 ± 3.60	26.29 ± 4.54	25.96 ± 4.97	0.819
Smoking, %	96 (34.4)	38 (40.0)	21 (29.2)	22 (31.9)	15 (34.9)	0.495
Hypertension, %	154 (55.2)	54 (56.8)	39 (54.2)	38 (55.1)	23 (53.5)	0.980
Diabetes mellitus, %	92 (33.0)	34 (35.8)	20 (27.8)	24 (34.8)	14 (32.6)	0.723
Hyperlipidemia, %	180 (64.5)	70 (73.7)	44 (61.1)	37 (53.6)	29 (67.4)	0.055
SBP, mm Hg	145.0 (121.0; 159.0)	147.0 (115.0; 163.0)	143.0 (121.0; 158.8)	142.0 (123.0; 154.0)	142.0 (124.0; 156.0)	0.962
DBP, mm Hg	77.0 (65.0; 88.0)	81.0 (65.0; 86.0)	74.5 (64.0; 90.0)	83.0 (66.0; 90.0)	75.0 (65.0; 90.0)	0.598
LVEF, %	53.3 (46.6; 60.7)	50.8 (42.8; 57.3)^b,c^	53.9 (45.2; 59.5)^c^	53.5 (48.7; 61.6)^c^	63.8 (52.2; 65.6)	< 0.001
LVEDVi, mL/m^2^	66.8 (57.9; 77.3)	75.9 (66.0; 82.2)^b,c^	72.0 (68.5; 80.9)^b,c^	59.9 (57.0; 65.1)	55.0 (51.9; 60.0)	< 0.001
LVESVi, mL/m^2^	30.7 (24.9; 37.5)	37.0 (30.8; 45.9)^b,c^	33.0 (29.9; 38.7)^b,c^	27.4 (23.3; 30.3)^c^	21.5 (18.8; 26.4)	< 0.001
Troponin T, ng/L	269.0 (154.0; 654.0)	632.0 (287.0; 1861.0)^b,c^	467.0 (239.0; 1469.5)^b,c^	216.0 (153.5; 321.0)^c^	85.0 (55.0; 102.0)	< 0.001
LGE, %	2.6 (0.0; 7.5)	7.5 (4.6; 11.2)^b,c^	4.4 (2.6; 8.4)^b,c^	0 (0)	0 (0)	< 0.001
Transmural LGE, %	39 (14.0)	34 (35.8)	5 (6.9)	0 (0)	0 (0)	< 0.001
Rest MBF, mL/min/g	1.2 (1.1; 1.2)	1.2 (1.1; 1.2)	1.2 (1.1; 1.3)	1.2 (1.1; 1.2)	1.2 (1.1; 1.2)	0.335
Stress MBF, mL/min/g	1.6 (1.5; 2.2)	1.4 (1.3; 1.6)^a,b,c^	2.1 (1.7; 2.3)^b,c^	1.6 (1.5; 1.7)^c^	2.4 (2.3; 2.6)	< 0.001
MPR	1.9 (1.8; 2.6)	1.7 (1.5; 1.9)^a,b,c^	2.5 (1.9; 2.7)^b,c^	1.9 (1.8; 1.9)^c^	2.8 (2.7; 3.1)	< 0.001

Values are mean ± standard deviation, *n* (%), or median (IQR). The chi-square test or Fisher’s exact test was used for categorical variables, analysis of variance for normally distributed variables, and the Kruskal–Wallis test for non-normally distributed continuous variables. Following significant omnibus tests, post hoc pairwise comparisons were carried out at the Bonferroni-corrected significance level of *p* = 0.008

Post hoc significant comparisons: ^a^ *p* < 0.05 versus LGE+/inducible ischemia−, ^b^ *p* < 0.05 versus LGE−/inducible ischemia+, ^c^ *p* < 0.05 versus LGE−/inducible ischemia−

*BMI* body mass index, *DBP* diastolic blood pressure, *LGE* late gadolinium enhancement, *LVEF* left ventricular ejection fraction, *LVEDVi* left ventricular end-diastolic volume index, *LVESVi* left ventricular end-systolic volume index, *MINOCA* myocardial infarction with nonobstructive coronary arteries, *MBF* myocardial blood flow, *MPR* myocardial perfusion reserve, *MRI* magnetic resonance imaging, *SBP* systolic blood pressure

The mean age of the study population was 56.35 ± 10.86 years, with females comprising over 55% of the cohort. There were no statistically significant differences in age, body mass index, gender and major cardiovascular risk factors among the different cardiac MRI feature subgroups. Among the 95 LGE+/inducible ischemia+ patients, 34 exhibited a transmural pattern of LGE, with a median LGE of 7.5%, which was significantly higher than in the other three groups. The normal cardiac MRI group (LGE−/inducible ischemia−) had significantly higher LVEF, higher stress MBF and MPR compared with the other three groups.

Additionally, we performed ROC curve analysis to determine the optimal cutoff value of MPR for predicting myocardial perfusion defects, with the optimal threshold identified using the Youden index. The ROC analysis showed that the area under the curve for MPR was 0.93 (95% CI: 0.898–0.963). The optimal MPR cutoff was 2.15, with a sensitivity of 79.1% and a specificity of 98.2%.

### Outcomes according to cardiac MRI phenotypes

After a median follow-up of 41.0 months (95% CI: 40.11–41.89), the overall all-cause mortality rate was 6.1%, and the composite endpoint (MACE) occurred in 11.5% of the entire cohort. The incidence of MACE at follow-up was significantly higher in LGE+/inducible ischemia+ MINOCA patients at 18.9%, compared to 2.3% in LGE−/inducible ischemia− patients (Table 2). Post hoc power analysis indicated that our sample size was sufficient to detect a 16.6-percentage-points difference in MACE between the LGE+/inducible ischemia+ and LGE−/inducible ischemia− groups (power = 81.8%, alpha = 0.05).

**Table 2:** Major adverse clinical events among patients with MINOCA according to cardiac MRI phenotypes

	Total (*N* = 279)	LGE+/inducible ischemia+ (*n* = 95)	LGE+/inducible ischemia− (*n* = 72)	LGE−/inducible ischemia+ (*n* = 69)	LGE−/inducible ischemia− (*n* = 43)	*p*-value
MACE	32 (11.5)	18 (18.9)	7 (9.7)	6 (8.7)	1 (2.3)	0.026
All-cause death	17 (6.1)	9 (9.5)	3 (4.2)	4 (5.8)	1 (2.3)	0.389
Reinfarction	10 (3.6)	4 (4.2)	1 (1.4)	5 (7.2)	0 (0)	0.176
Stroke	1 (0.4)	0 (0)	0 (0)	0 (0)	1 (2.3)	0.154
Heart failure	20 (7.2)	13 (13.7)	6 (8.3)	1 (1.4)	0 (0)	0.003

Values are *n* (%)

*MRI* magnetic resonance imaging, *LGE* late gadolinium enhancement, *MACE* major adverse cardiovascular events, *MINOCA* myocardial infarction with nonobstructive coronary arteries

**Fig. 5:**
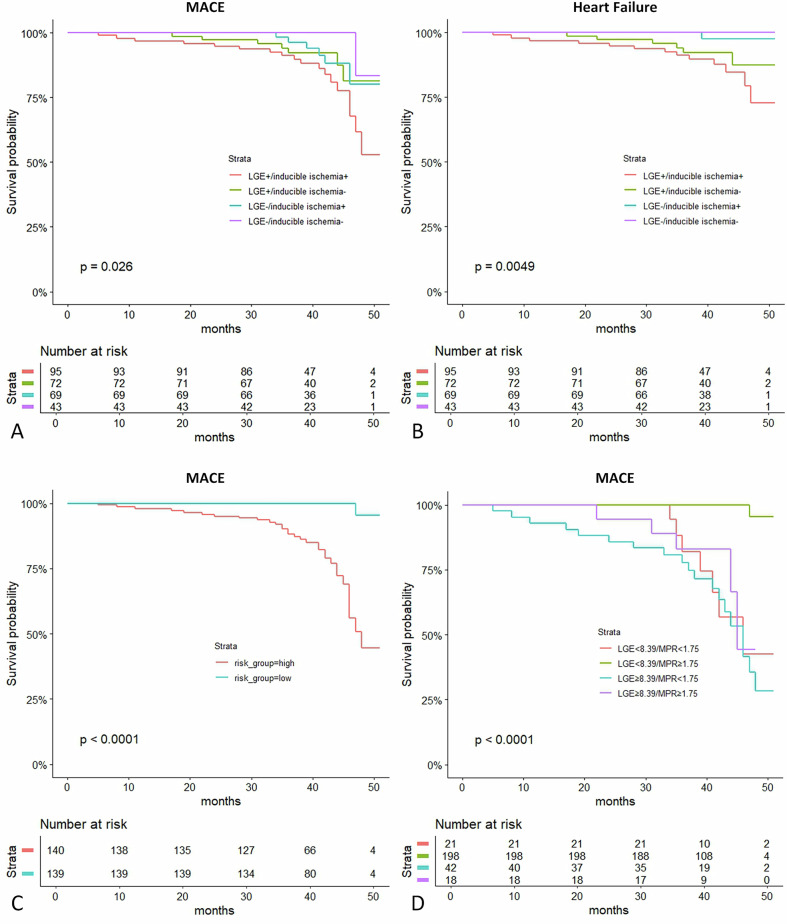
Kaplan–Meier curves for MACE and heart failure. **A** LGE+/inducible ischemia+ patients exhibited significantly higher MACE incidence versus LGE−/inducible ischemia− patients. **B** Heart failure occurrence significantly greater in LGE+/inducible ischemia+ patients than in others. **C** Kaplan–Meier survival analysis based on the median of predictive model score. **D** Kaplan–Meier survival analysis based on the optimal cutoff value of LGE and MPR. LGE, late gadolinium enhancement; MACE, major adverse cardiovascular events; MPR, myocardial perfusion reserve

No significant differences were observed between groups in terms of all-cause mortality, reinfarction, or stroke (Table 2). However, heart failure occurred more frequently in LGE+/inducible ischemia+ patients with MINOCA (13.7%) compared with LGE−/inducible ischemia+ patients (8.3%) and LGE−/inducible ischemia− patients (1.4%) (*p* = 0.003). Kaplan–Meier estimates for MACE and heart failure occurrence are shown in Fig. 5. The results of the pairwise comparisons for MACE are presented in Supplementary Table S1.

In addition, patients who developed MACE demonstrated lower LVEF, reduced stress MBF and MPR, elevated LVESVi and cardiac troponin T levels, and greater LGE volume, along with a higher prevalence of transmural LGE pattern, compared to those without MACE (Table 3).

**Table 3:** Characteristics of patients with and without MACE

	MACE (*n* = 32)	No MACE (*n* = 247)	*p*-value
Age, years	54.31 ± 10.34	56.62 ± 10.92	0.695
Sex (female)	21 (65.6)	133 (53.8)	0.207
SBP, mm Hg	145.0 (113.5; 162.8)	145.0 (122.0; 157.0)	0.839
DBP, mm Hg	78.5 (63.5; 88.0)	77.0 (65.0; 88.0)	0.943
LVEF, %	38.6 (35.2; 51.6)	54.5 (48.7; 61.6)	< 0.001
LVEDVi, mL/m^2^	70.1 (57.1; 76.6)	66.3 (58.0; 77.5)	0.625
LVESVi, mL/m^2^	39.2 (32.4; 47.4)	29.9 (24.6; 36.2)	< 0.001
Troponin T, ng/L	1412.5 (499.0; 3936.3)	254.0 (151.0; 581.0)	< 0.001
LGE, %	9.6 (7.7; 25.6)	2.4 (0.0; 6.2)	< 0.001
Transmural LGE, %	18 (56.3)	21 (8.5)	< 0.001
Rest MBF, mL/min/g	1.1 (1.1; 1.2)	1.2 (1.1; 1.3)	0.019
Stress MBF, mL/min/g	1.4 (1.3; 1.5)	1.7 (1.5; 2.2)	< 0.001
MPR	1.5 (1.4; 1.7)	1.9 (1.8; 2.6)	< 0.001

Values are mean ± standard deviation, *n* (%), or median (IQR)

*DBP* diastolic blood pressure, *LGE* late gadolinium enhancement, *LVEF* left ventricular ejection fraction, *LVEDVi* left ventricular end-diastolic volume index, *LVESVi* left ventricular end-systolic volume index, *MACE* major adverse cardiovascular events, *MINOCA* myocardial infarction with nonobstructive coronary arteries, *MBF* myocardial blood flow, *MPR* myocardial perfusion reserve, *SBP* systolic blood pressure

### Incremental value of MPR for predicting MACE

Multivariate Cox regression analysis showed that LGE (HR: 1.108, 95% CI: 1.047–1.172, *p* < 0.001) and MPR (HR: 0.062, 95% CI: 0.007–0.524, *p* = 0.011) were identified as independent predictors of MACE in patients with MINOCA (Table 4). Based on the median risk score calculated from the multivariable Cox model using R software, patients were divided into high- and low-risk groups. Kaplan–Meier survival analysis revealed that patients in the high-risk group had lower survival probabilities than those in the low-risk group (*p* < 0.001) (Fig. 5C).

**Table 4:** Independent predictors of MACE in patients with MINOCA

	Univariate regression			Multivariate regression		
	HR	95% CI	*p*-value	HR	95% CI	*p*-value
Age, years	0.981	0.948–1.015	0.261			
Female	1.705	0.820–3.543	0.153			
Smoking	0.800	0.378–1.690	0.558			
Hypertension	1.271	0.627–2.575	0.506			
Diabetes mellitus	0.642	0.277–1.488	0.301			
Hyperlipidemia	0.600	0.300–1.201	0.149			
LVEF, %	0.834	0.791–0.880	< 0.001			
LVEDVi, mL/m^2^	1.009	0.980–1.038	0.557			
LVESVi, mL/m^2^	1.103	1.062–1.145	< 0.001			
Troponin T, ng/L	1.000	1.000–1.001	< 0.001			
LGE, %	1.185	1.141–1.230	< 0.001	1.108	1.047–1.172	< 0.001
Trasmural LGE	10.069	4.996–20.292	< 0.001			
Rest MBF, mL/min/g	0.023	0.000–1.136	0.058			
Stress MBF, mL/min/g	0.008	0.001–0.060	< 0.001			
MPR	0.003	0.000–0.023	< 0.001	0.062	0.007–0.524	0.011

*LGE* late gadolinium enhancement, *LVEF* left ventricular ejection fraction, *LVEDVi* left ventricular end-diastolic volume index, *LVESVi* left ventricular end-systolic volume index, *MACE* major adverse cardiovascular events, *MINOCA* myocardial infarction with nonobstructive coronary arteries, *MBF* myocardial blood flow, *MPR* myocardial perfusion reserve

Patients were stratified into 4 groups based on the optimal cutoff value derived from ROC curve analysis (8.39 for LGE and 1.75 for MPR). Kaplan–Meier survival analysis demonstrated that patients with LGE ≥ 8.39 and MPR < 1.75 exhibited the highest long-term risk of MACE (*p* < 0.001) (Fig. 5D). Critically, among patients with limited LGE (< 8.39), those with MPR ≥ 1.75 showed significantly better clinical outcomes than those with MPR < 1.75 (*p* < 0.001) (Supplementary Table S2).

### Inter-observer and intra-observer variability

The reproducibility of cardiac MRI parameters was excellent, with ICC values of 0.960–0.999 (*p* < 0.001) (Supplementary Table S3).

## Discussion

This study establishes the prognostic value of stress cardiac MRI and demonstrates its utility for risk stratification in MINOCA patients through phenotype-based subgroup analysis. Our main findings indicate that both LGE and MPR independently predict clinical outcomes. Importantly, MPR offers incremental prognostic value by further stratifying risk among patients with limited LGE. These results highlight the importance of incorporating stress cardiac MRI early in the diagnostic workup and prognostic assessment for the comprehensive management of MINOCA.

We stratified patients according to the presence or absence of LGE and inducible ischemia on cardiac MRI. This dual-phenotype classification aligns with the current pathophysiological understanding of MINOCA and is supported by clinical evidence. MINOCA is essentially a heterogeneous syndrome encompassing a variety of potential etiologies. The predominant mechanism underlying myocardial injury in MINOCA is ischemic [22]. LGE imaging is a sensitive tool for tissue characterization. The specific pattern and distribution of LGE allow differentiation between ischemic and non-ischemic MINOCA [23]. Furthermore, coronary microvascular dysfunction, involving both functional and structural abnormalities attributed to endothelial and vascular smooth muscle cell dysfunction, is prevalent in a substantial proportion of MINOCA patients [7]. Mauricio et al [24] found that abnormal stress perfusion matched the location of myocardial scar in only 75% of cases. In our study population, we identified patients exhibiting stress perfusion abnormalities in the absence of LGE. In the present study, we further identified a diagnostic threshold of MPR ≤ 2.15 for predicting stress-induced perfusion defects, with an area under the curve of 0.93, sensitivity of 79.1%, and specificity of 98.2%. This threshold is similar to prior investigations demonstrating that MPR values ≤ 2.19 reliably identify coronary microvascular dysfunction in patients with angina and nonobstructive coronary artery disease [25]. This classification framework aids in differentiating MINOCA subtypes according to underlying etiology, forming a basis for risk stratification and guiding individualized treatment.

During follow-up, recurrent myocardial infarction occurred in 3.6% of the cohort. The incidence of reinfarction was numerically higher in the LGE−/inducible ischemia+ group than in the LGE+/inducible ischemia− group, although this difference did not reach statistical significance. This observation may be attributable to the more severe coronary microvascular dysfunction, which is characterized by a lower MPR. The most common microvascular causes of MINOCA are the obliteration of coronary microcirculation linked to thromboembolism or microvascular spasm [26]. Recent studies reported that coronary stenosis progressed in about half of patients with reinfarction after MINOCA [27, 28]. Additionally, microvascular spasm occurs in approximately 16–31% of MINOCA patients [29–31]. In a cohort of 40 female MINOCA patients, 63% exhibited stress-induced perfusion abnormalities on cardiac MRI, potentially indicating the presence of diffuse microvascular disease [24]. These findings suggested that coronary microvascular dysfunction is emerging as a major cause of MINOCA and may influence clinical outcomes. Microvascular dysfunction in MINOCA patients can induce stress-induced myocardial hypoperfusion, leading to myocardial ischemia and consequently elevating the risk of reinfarction and MACE.

In our study, both LGE and MPR independently emerged as strong predictors of outcome. The presence and transmural pattern of LGE were associated with increased all-cause death and MACE, as previously reported [23, 25, 26]. Our findings align with previous analyses establishing that reductions in MPR using cardiac MRI were independently associated with adverse outcomes [32].

This study further demonstrates the incremental predictive value of MPR in patients with MINOCA. Patients with extensive LGE but preserved MPR experienced relatively favorable outcomes, whereas those with limited LGE but impaired MPR showed a higher incidence of MACE. These observations substantiate the evidence that microvascular dysfunction represents a potential mechanism in MINOCA and is associated with adverse cardiovascular outcomes [33–35]. Reynolds et al [36] revealed that, among 116 female patients with MINOCA, 19 of 24 patients with cardiac MRI evidence of regional injury but no LGE had a culprit lesion identified by coronary optical coherence tomography. This finding could be explained by functional microvascular dysfunction without structural myocardial damage or by a short duration of the ischemic injury, which results in less cell necrosis and, therefore, no detectable gadolinium accumulation. Severe MPR impairment reflects microvascular dysfunction. In cases with limited LGE but reduced MPR, persistent microvascular dysfunction can precipitate recurrent ischemia, myocardial stunning, and eventual disease progression, ultimately driving adverse outcomes.

Our classification strategy, based on LGE and inducible ischemia, differs from the approach used by Bergamaschi et al [37], which utilized LGE and abnormal mapping. The difference in grouping methods stems from the fact that the MINOCA patients enrolled in our study were evaluated during the non-acute phase. For such patients, mapping techniques demonstrate reduced efficacy for detecting early myocardial edema.

## Limitations

This study has several limitations that warrant consideration. First, the retrospective and single-center design carries inherent limitations, such as selection bias and unmeasured confounding factors, which may restrict the generalizability of the findings. Although the sample size is adequate for the comparison between the LGE+/inducible ischemia+ and LGE−/inducible ischemia− groups, it may still have been underpowered for other subgroup analyses. Second, we did not perform additional invasive plaque imaging (such as optical coherence tomography or intravascular ultrasound) or functional tests, as these techniques are costly, carry procedural risks, and are time-consuming. Third, the total MPR values in our study were calculated globally across the entire myocardium, and the inclusion of scar regions may partially attenuate the measured MPR values. Additionally, the MPR thresholds derived from this study may not be applicable when using a different imaging system and should not be directly extrapolated to other protocols. Finally, the research did not take into consideration differences in pharmacological or surgical treatment approaches. Subtle differences in treatment adherence, intensity of therapeutic management, or differences in the use of medications could influence event risk. Future studies should focus on analyzing the effects of these treatment variations on outcomes, as they are possible confounding factors that might have a major impact on prognosis.

## Conclusions

Our study demonstrates that both LGE and MPR, quantitatively detected by stress cardiac MRI, offer critical prognostic insights for risk stratification in MINOCA. MPR provided independent prognostic value by distinguishing patient outcomes even when LGE was limited, confirming the crucial role of myocardial microvascular dysfunction. Our findings may offer a preliminary framework that could potentially aid in identifying high-risk MINOCA patients and guide future research on therapeutic strategies.

## Supplementary information

ELECTRONIC SUPPLEMENTARY MATERIAL

## Data Availability

The data that support the findings of this study are available from the corresponding author upon request.

## Abbreviations

AIF: Arterial input function
LGE: Late gadolinium enhancement
LVEDVi: Left ventricular end-diastolic volume index
LVEF: Left ventricular ejection fraction
LVESVi: Left ventricular end-systolic volume index
MACE: Major adverse cardiovascular events
MBF: Myocardial blood flow
MI: Myocardial infarction
MINOCA: Myocardial infarction with nonobstructive coronary artery
MPR: Myocardial perfusion reserve
MRI: Magnetic resonance imaging
