# Supplementary material for: Prognosis and risk stratification in first-presentation myocardial infarction with nonobstructive coronary arteries using stress cardiac MRI
Source: Insights Imaging. 2026 May 29;17:148. doi: 10.1186/s13244-026-02316-2 (PMC13221553; doi:10.1186/s13244-026-02316-2)
Supplement: Supplementary file 1 — ELECTRONIC SUPPLEMENTARY MATERIAL [file 13244_2026_2316_MOESM1_ESM.pdf]

Prognosis and Risk Stratification in First-Presentation Myocardial Infarction with Nonobstructive Coronary Arteries Using Stress Cardiac MRI

ELECTRONIC SUPPLEMENTARY MATERIAL

Supplementary Tables

Table S1. Pairwise comparisons of MACE using the log-rank test.

	LGE+/inducible ischemia+		LGE+/inducible ischemia-		LGE-/inducible ischemia+		LGE-/inducible ischemia-	
	χ^2	P value	χ^2	P value	χ^2	P value	χ^2	P value
LGE+/inducible ischemia+	-	-	2.46	0.117	3.019	0.082	6.739	0.009
LGE+/inducible ischemia-	2.46	0.117	-	-	0.048	0.827	2.373	0.123
LGE-/inducible ischemia+	3.019	0.082	0.048	0.827	-	-	1.935	0.164
LGE-/inducible ischemia-	6.739	0.009	2.373	0.123	1.935	0.164	-	-

LGE, late gadolinium enhancement; MACE, major adverse cardiovascular events.

Table S2. Pairwise comparisons of MACE using the log-rank test.

	Group 1		Group 2		Group 3		Group 4	
	χ^2	P value	χ^2	P value	χ^2	P value	χ^2	P value
Group 1	-	-	61.204	<0.001	0.433	0.510	0.098	0.754
Group 2	61.204	<0.001	-	-	79.427	<0.001	44.93	<0.001
Group 3	0.433	0.510	79.427	<0.001	-	-	0.76	0.383
Group 4	0.098	0.754	44.93	<0.001	0.76	0.383	-	-

Group 1: LGE <8.39/MPR <1.75
Group 2: LGE <8.39/MPR ≥1.75
Group 3: LGE ≥8.39/MPR <1.75
Group 4: LGE ≥8.39/MPR ≥1.75
LGE, late gadolinium enhancement; MACE, major adverse cardiovascular events; MPR, myocardial perfusion reserve.

Table S3.

Parameters	Intra-observer		Inter-observer	
	ICC	95% CI	ICC	95% CI
LVEF (%)	0.986	0.971-0.993	0.975	0.949-0.988
LVEDVi (mL/m^2)	0.998	0.995-0.999	0.992	0.983-0.996
LVESVi (mL/m^2)	0.996	0.992-0.998	0.992	0.983-0.996
LGE (%)	0.999	0.997-0.999	0.996	0.992-0.998
Rest MBF (mL/min/g)	0.964	0.927-0.983	0.960	0.917-0.981
Stress MBF (mL/min/g)	0.986	0.970-0.993	0.981	0.960-0.991

ICC, intraclass correlation coefficient; LGE, late gadolinium enhancement; LVEF, left ventricular ejection fraction; LVEDVi, left ventricular end-diastolic volume index; LVESVi, left ventricular end-systolic volume index; MBF, myocardial blood flow; MPR, myocardial perfusion reserve.
